# Beyond the Procedure: Non-Clinical Complaints and Gender Disparity Dominate One-Star Yelp Reviews of Pain Physicians

**DOI:** 10.1007/s11916-026-01471-x

**Published:** 2026-03-11

**Authors:** Andrew Owens, Jaden Poulter, Blaze Borowski, Sarang Koushik, Omar Viswanath, Matthew Smith, Paul Kang, Brian Wilhelmi

**Affiliations:** 1 Creighton University School of Medicine, 3rd year Medical Students, Phoenix, AZ USA; 2 Department of Anesthesiology and Perioperative Medicine, Mayo Clinic, Phoenix, AZ USA; 3 Department of Anesthesiology, Creighton University School of Medicine, Phoenix, AZ USA; 4 St. George’s University School of Medicine, 3rd Year Medical Student, St. George’s, Grenada; 5 Creighton University, School Statistician, Phoenix , AZ USA; 6 Barrow Department of Neuroanesthesiology, Faculty Mentor with Creighton School of Medicine, Phoenix, AZ USA

**Keywords:** Pain Medicine, Quality and Performance Improvement, Practice Management and Organization, Anesthesiologist

## Abstract

**Background:**

Yelp.com allows patients to review their experiences. Few studies have focused on dissatisfaction in pain medicine. This study aims to characterize one-star Yelp reviews of anesthesiology-trained pain physicians to better understand the scope and nature of extreme patient dissatisfaction.

**Results:**

Non-clinical issues comprised the most complaints (*n* = 927; 70%), while clinical concerns comprised 391 (30%). Office staff communication topped non-clinical complaints (*n* = 200). Unsatisfactory results (*n* = 90) topped clinical concerns. Eastern cities like Philadelphia, PA (53%) had the most one-star reviews, whereas Western cities such as Los Angeles, CA (21%) had the lowest. Solo practices were associated with a higher rate of one-star reviews (33%) than group practices (28%). Gender-based analysis showed that female-only practices received significantly more one-star reviews (mean = 12.17) compared to male-only (mean = 2.90) and mixed-gender (mean = 7.00) practices. Poisson regression analysis indicated a higher relative risk of one-star reviews for female providers (RR = 1.00 [ref]), with reduced risk for male providers (RR = 0.58) and increased risk for mixed-gender practices (RR = 1.40; all *p* < 0.001).

**Conclusion:**

Patient dissatisfaction is most frequently due to non-clinical experiences. Regional factors, practice type, and physician gender demonstrated significant associations with patterns of patient dissatisfaction. Eastern cities exhibited higher rates of negative reviews. Solo practitioners appeared more susceptible to critical feedback. Female providers seemed to have a disproportionate number of one-star ratings. These trends may reflect underlying systemic and implicit biases and demonstrate vulnerabilities within specific practice models.

## Introduction

Pain physicians are a highly sought-after physician specialty in the United States [1]. Given the high demand for pain specialists, patients often have multiple options to choose from when selecting a physician. In this modern age, patients are empowered to seek information and be their own advocates in selecting physicians to treat them. Whether this is word-of-mouth or referrals from trusted physicians, what patients hear about physicians is often largely influential on whether they decide to see them as a provider or not [2]. 

Over the past decade, a new form of physician review has gained increasing popularity. Yelp.com, namely, has been a go-to for many patients to see what experiences have been for other people in a similar health position [3, 4]. Whether it has been for orthopedic surgery, ophthalmic surgery, or pain physicians, Yelp.com has provided a way for patients to see what other people think about the doctor before visiting themselves [5–8]. 

From the physician’s perspective, this poses both benefits and challenges to practice. Pain physicians are specifically trained to address both chronic and acute manifestations of pain, utilizing both procedural and pharmacological treatments [9]. These scientific approaches are easily reportable, allowing physicians to be compared on patient outcomes using evidence-based treatments as the reference point. However, with the passage of the Affordable Care Act in 2010, along with the Centers for Medicare and Medicaid services (CMS) pushing for increased data release, providers were able to be compared in a significantly more transparent fashion, with patient outcomes and satisfaction highlighted [5, 10]. 

This preference for anecdotal evidence is evident in the widespread use of online physician rating platforms such as Yelp.com, which has become the most frequently consulted resource for patients seeking physician reviews despite the availability of more objective quality indicators [4, 11]. In fact, Yelp.com was superior to other government sponsored methods of reporting patient outcomes and physician information, such as CMS‘s hospital compare site [12]. With this largely crowd-sourced data, a huge variety of reviews is available. These reviews, positive or negative, can influence a potential future patient’s decision of whether they want to see a certain provider or not [13]. Despite this, there exists no study in the literature that has characterized the factors associated with extremely negative reviews for pain physicians. The purpose of the study was to characterize extremely negative reviews of pain physicians on Yelp.com.

## Materials and Methods

We conducted a retrospective review of publicly available Yelp.com reviews to assess patient-reported complaints about board-certified pain physicians. The search terms *“Pain Doctor”* and *“Anesthesiologist”* were applied to Yelp.com in the 10 most populous U.S. cities (New York, Los Angeles, Chicago, Houston, Phoenix, Philadelphia, San Antonio, San Diego, Dallas, and Jacksonville). For each city, the top 10 listed pain physicians were identified. Accreditation as board-certified anesthesiologists specializing in pain management was confirmed through secondary sources, including Doximity, AAPM, physician websites, Healthgrades, Vitals, and Angie’s List.

Only one-star reviews were included; reviews with ≥ 2 stars were excluded. Identifying information (reviewer names, profiles) was not recorded. Reviews were coded using a structured framework that categorized complaints into 13 clinical and 13 non-clinical domains. Complaints were further classified as procedural or non-procedural. Descriptive statistical analysis was performed to calculate means, rates, and rate ratios.

## Results

**Fig. 1:**
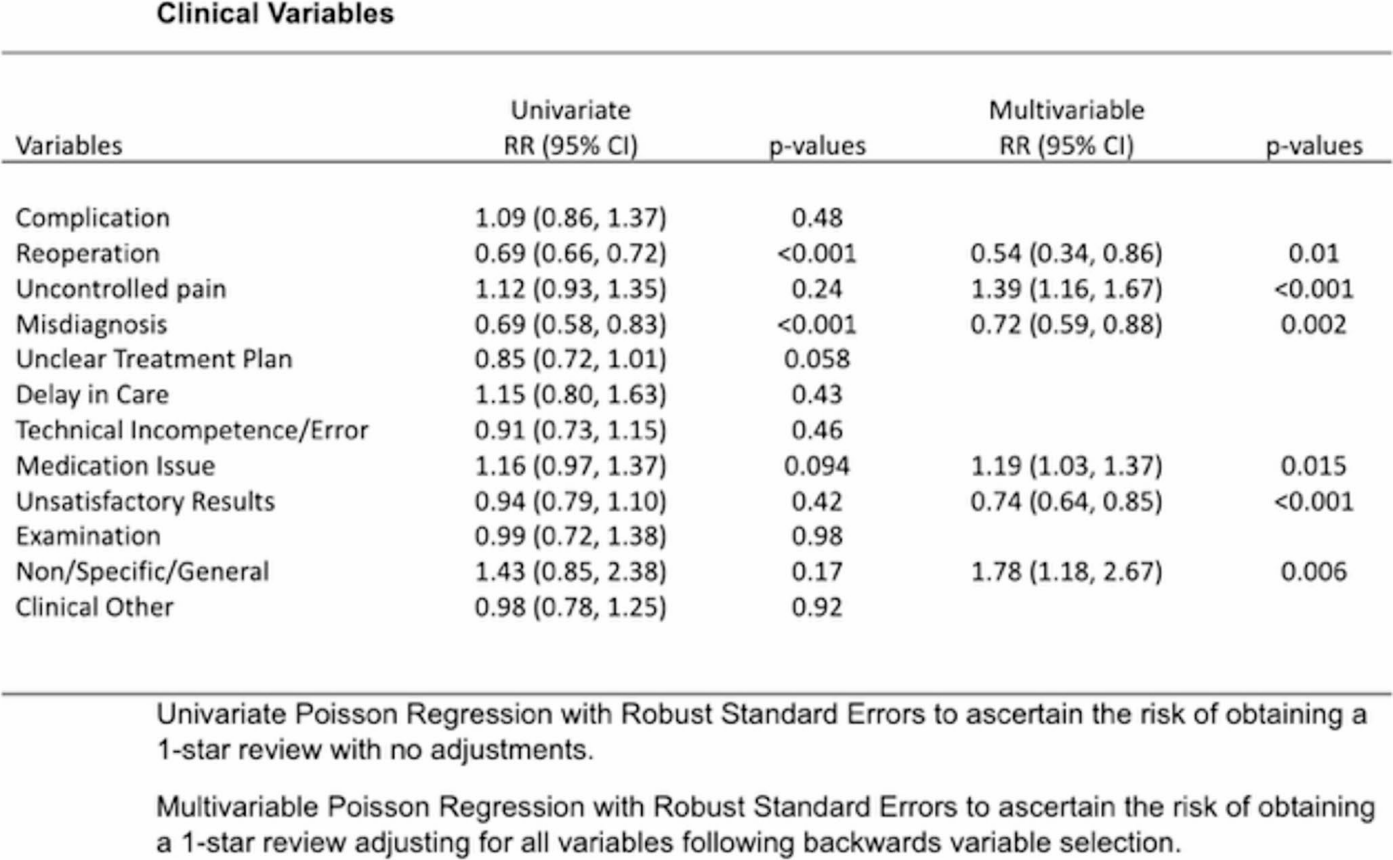
– Clinical Variables with univariate and multivariate analysis and respective relative risk of receiving 1-star review

A total of 1415 Yelp reviews were analyzed. When stratifying online reviews by clinical and non-clinical correlations, we see that there were several factors significantly associated with the likelihood of a 1-star review. Using univariate analysis, unsatisfactory results decreased the risk of a one star review (RR = 0.74, 95% CI 0.64–0.85, *p* < 0.001). Factors that significantly predicated 1-star reviews were uncontrolled pain (RR = 1.39, 95% CI 1.16–1.67, *p* < 0.001), cost/billing/insurance issues (RR = 1.13, 95% CI 1.01–1.28, *p* = 0.033), and facilities/office environment (RR = 0.81, 95% CI 0.66–0.99, *p* = 0.042) (Fig. 1).

**Fig. 2:**
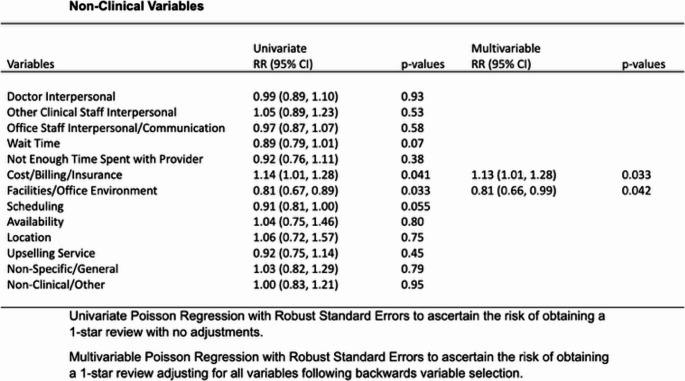
– Non-Clinical variables with univariate and multivariate analysis and respective relative risk of receiving 1-star review

When adjusting for all variables and using a multivariate regression analysis, it was confirmed that uncontrolled pain (RR = 1.39, 95% CI 1.16–1.67, *p* < 0.001) and misdiagnosis (RR = 0.72, 95% CI 0.59–0.88, *p* = 0.002) were significant contributors to 1-star reviews in the clinical category (Fig. 2). Looking at non-clinical factors, office staff communication (RR = 1.78, 95% CI 1.18–2.67, *p* = 0.006) and cost/billing/insurance issues (RR = 1.13, 95% CI 1.01–1.28, *p* = 0.033) were most strongly associated with 1-star reviews.

**Fig. 3:**
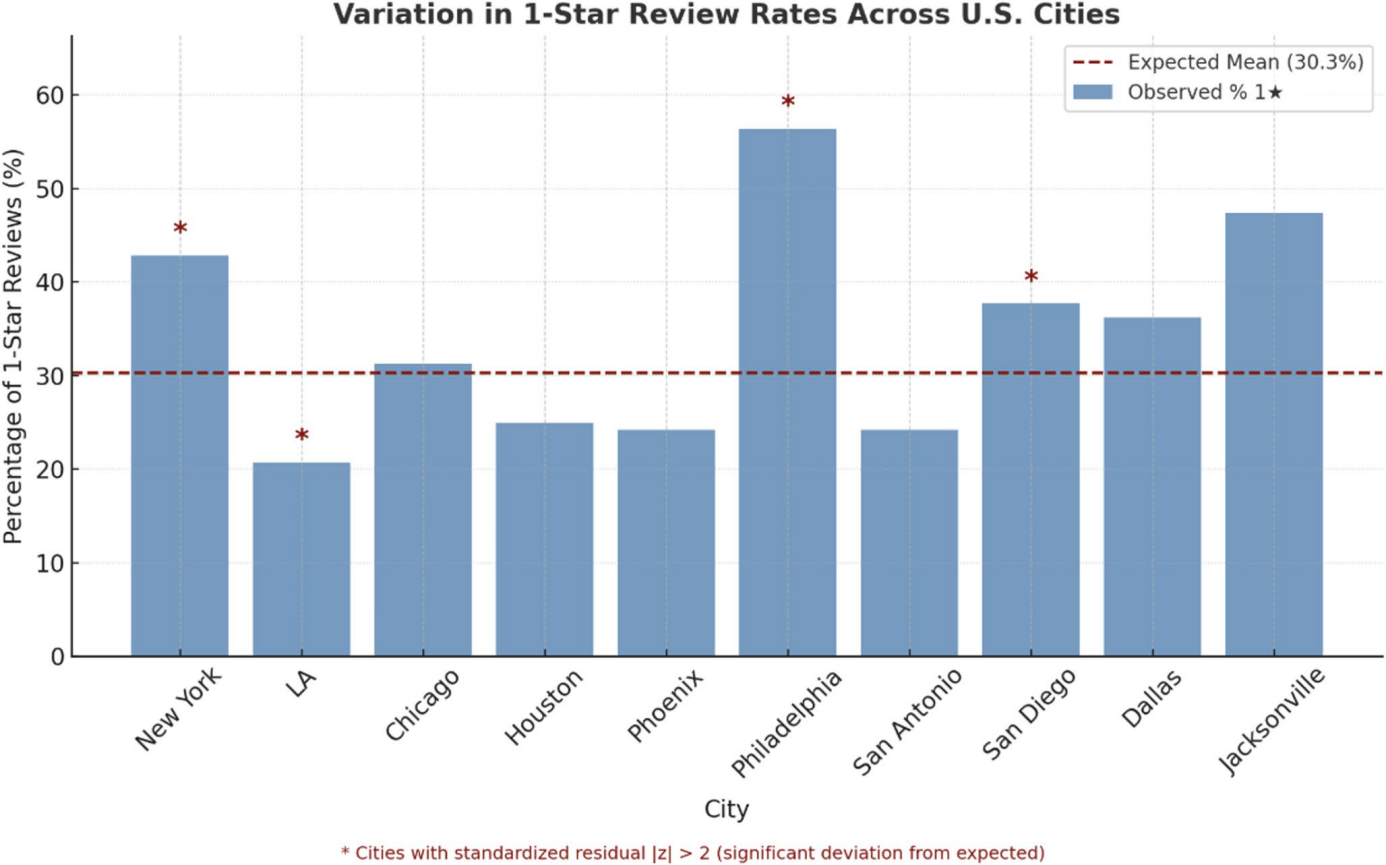
– Variability in rates of receiving 1-star reviews across the 10 most populous US cities

**Fig. 4:**
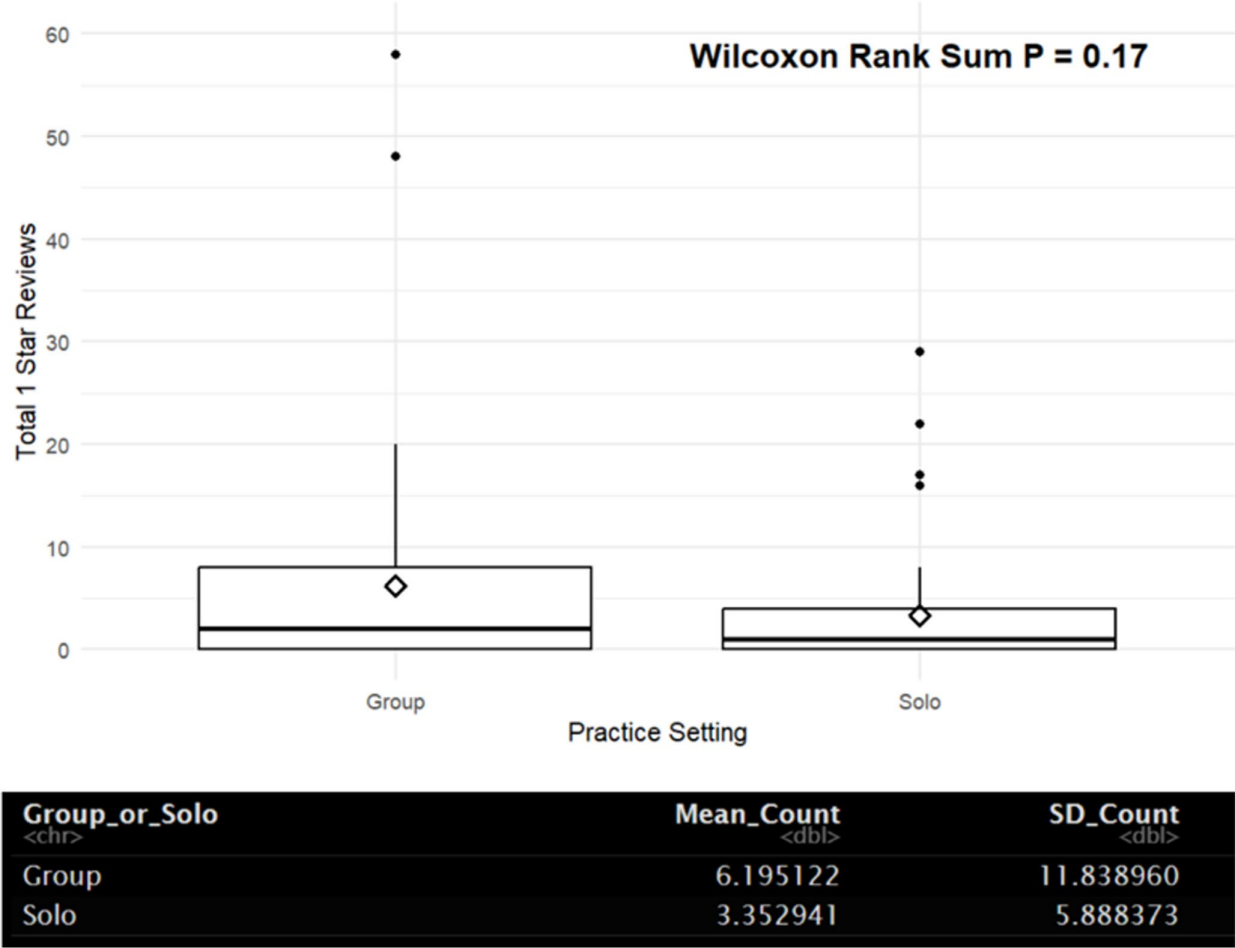
– Wilcoxon Rank Sum analysis of practice type; group practice or solo practice

Reviews were also stratified by geographic location and practice type in terms of group practice or solo practice (Fig. 3). Of the 1415 reviews analyzed, 30% (425) were 1-star. Based on geographical location, the cities with the highest percentage of 1-star reviews were Philadelphia, PA (53%), Jacksonville, FL (47%), and New York, NY (43%). Cities with the lowest percentage of 1-star reviews were Los Angeles, CA (21%), San Antonio, TX (22%), and Phoenix, AZ (28%) (Fig. 4).

Of the clinics that comprised reviews in this study, 55% were group practices. These group practices received 903 reviews, 28% of which were 1-star. Solo physicians obtained 512 reviews, 33% of which were 1-star reviews.

**Fig. 5:**
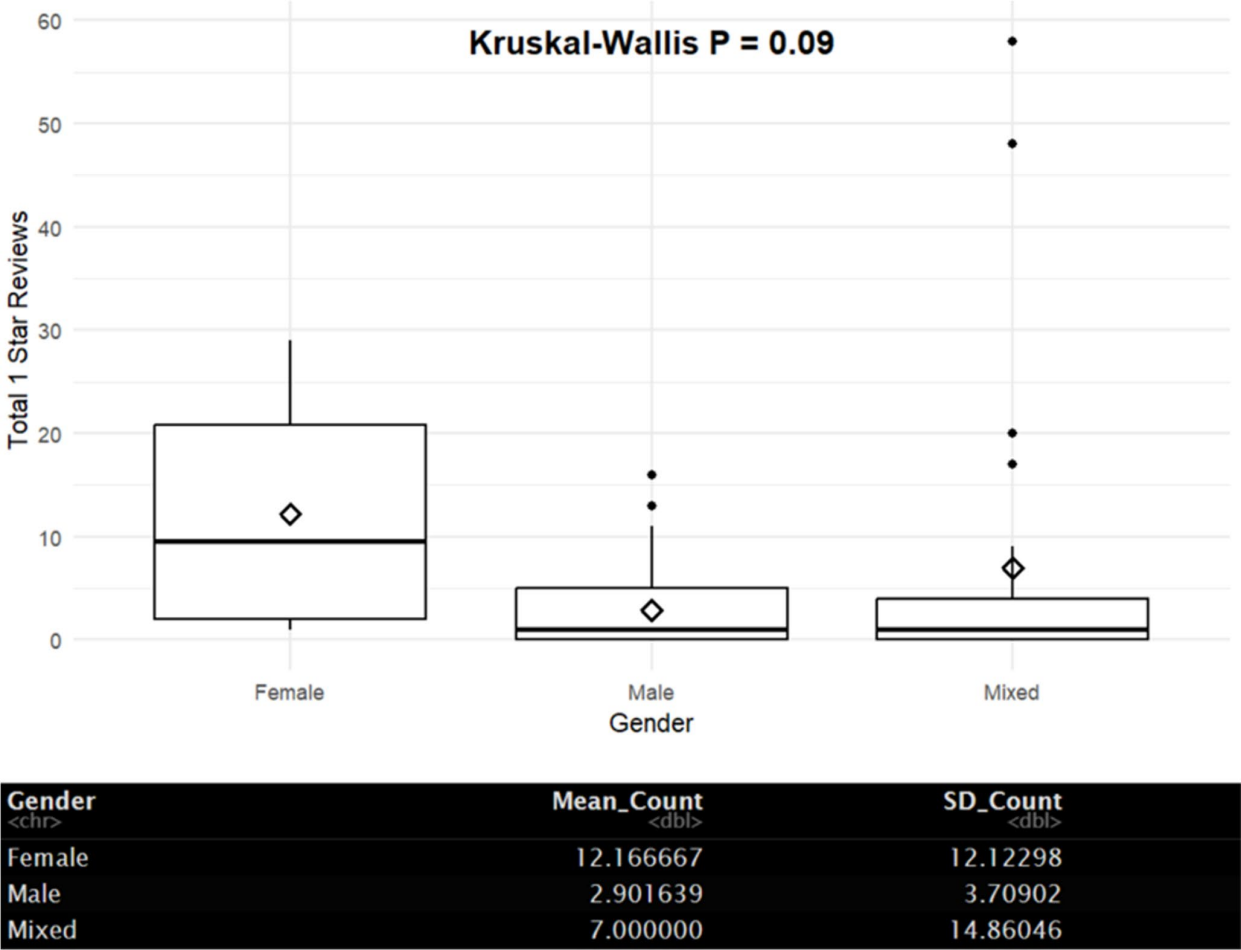
– Kruskal-Wallis analysis of practice by gender

Finally, reviews were examined after stratifying pain physicians by gender. Using a Kruskal-Wallis analysis, female-only pain physician practices received more 1-star reviews in general when compared to male-only and mixed gender practices (Fig. 5). Female-only pain physicians received a mean of 12.17 1-star reviews compared to 2.90 in male-only practices and 7.00 in mixed gender practices (*p* = 0.09). The likelihood of receiving a 1-star review in female-only practices when compared to male-only and mixed gender practices was 96.1% (*p* < 0.001). Interestingly, the likelihood of receiving a 1-star review as a male-only and mixed gender practice was 26.6% and 71.4%, respectively (*p* < 0.001).

A Poisson regression using female-only practices as a reference point gave a relative risk of receiving 1-star reviews as a male-only or mixed gender practice. In male-only practices, there was a decreased relative risk of receiving a 1-star review compared to female-only practices (RR = 0.58, 95% CI 0.58, 0.58, *p* < 0.001). As for mixed gender practices, there was an increased relative risk for 1-star of reviews compared to female-only practices (RR = 1.40, 95% CI 1.39, 1.40, *p* < 0.001).

## Discussion

This study aims to offer a comprehensive analysis of one-star Yelp reviews specifically directed at board certified anesthesiology-trained Pain physicians. The findings presented here offer a clear claim that non-clinical factors are the more dominant drivers leading to extreme patient frustration and dissatisfaction, even more than clinical outcomes.

Non-clinical factors lead to more one-star reviews than clinical factors. The most frequently cited issues were non-clinical in nature, with poor communication from office staff, generalized billing complaints, and inefficient office environments being the three major complaints seen in negative reviews. These results align with findings from other specialties, such as orthopedics and dermatology, where non-clinical complaints similarly outweighed concerns about clinical care. (3–7) Because pain medicine often offers long-term relationships and multiple visits, negative encounters with the front-desk or administrative miscommunications may carry a heavy emotional impact for patients already living with chronic pain and discomfort.

Clinical complaints still contribute to one-star reviews, especially when pain is unresolved. Although less common, clinical concerns are noted in many 1-star reviews. The two most frequent clinical complaints observed are uncontrolled pain and unsatisfactory results. The persistence of pain is arguably the core issue patients seek to resolve, suggesting that when treatment fails to meet expectations even clinically appropriate care may be received in a negative manner. These findings highlight the importance of establishing realistic expectations and maintaining transparent communication throughout the treatment process.

Geographic and practice structure have unique implications in the tendency a provider may have to receive 1-star reviews. As noted in the results, east coast cities such as Philadelphia and New York had a disproportionately high rate of receiving 1-star Yelp reviews compared to western cities like Los Angeles and Phoenix. Cultural expectations or regional differences in healthcare delivery may contribute to this trend, however, these findings warrant further investigation into location-specific factors that may be at play particularly for pain physicians practicing on the east coast.

Likewise, solo providers rather than those in a group were more frequently associated with 1-star reviews. This may reflect a limited capacity to handle patient grievances. Group practice may benefit from shared resources, internal quality control, and improved communication systems to overcome or resolve patient dissatisfaction (16).

Gender disparities are strikingly apparent in 1-star review ratings. Perhaps the most concerning finding was the higher rate of 1-star reviews given to female pain physicians. This is most likely due to an implicit bias influencing patient perception and evaluation. Prior research has observed gender-based disparities in multiple fields requiring advanced education. (14-15) The findings in this study contribute to the concern that female providers may face a disproportionate level of scrutiny compared to male counterparts.

While this study offers important insights, there are limitations worth noting. Yelp reviews reflect the opinions of a self-selecting group of patients and may not capture the broader patient population. The anonymity of the reviews prevents analysis based on demographics or specific clinical contexts, limiting the ability to draw conclusions about which patient groups are most affected. Additionally, gender analysis was based on publicly available practice listings. This may not reflect the true provider composition.

## Conclusions

Future research could expand on this work by comparing across multiple review platforms, analyzing a wider range of ratings, or incorporating direct patient surveys to validate these trends. Further study is also needed to better understand how implicit bias and regional differences shape patient perceptions in pain medicine.

Ultimately, understanding the drivers for extreme dissatisfaction may inform strategies to improve the patient experience and physician reputability. Targeting improvement in front office communication, billing transparency, and expectation-setting may yield the greatest benefit in reducing the risk of negative reviews. Addressing these concerns, as well as those discussed above, may be just as important as clinical outcomes in improving public perception and patient-physician trust.

## Key Reference

- Kraetschmer, N., Sharpe, N., Urowitz, S. and Deber, R.B. (2004), How does trust affect patient preferences for participation in decision-making?. Health Expectations, 7: 317-326. 10.1111/j.1369-7625.2004.00296.x
  - ○ This study examined trust in physicians as it relates the desire of a patient to participate in an active role in their care. The foundation of our study ultimately examines how much a negative review might impact the implicit or initially perceived credibility a patient may place in a doctor and consequently their future care. Thus, showing that high trust led to a more active role in their own care relates to how a negative rating can alter the patient’s perception/trust of the physician.

- Arthur JR, Etzioni D, Schwartz AJ. Characterizing extremely negative reviews of total joint arthroplasty practices and surgeons on yelp.com. Arthroplast Today. 2019;5(2):216-220. Published 2019 Apr 10. doi:10.1016/j.artd.2019.02.009
  - ○ This study was an important reference in how our methodology was to be set, as well as how the data we collected could be stored and examined. This study examined patient satisfaction via Yelp reviews in orthopedic surgeons, serving as an example for the most efficient way to go about this study.

## Data Availability

No datasets were generated or analysed during the current study.
